# Quantitative MRI in early intervertebral disc degeneration: T1rho correlates better than T2 and ADC with biomechanics, histology and matrix content

**DOI:** 10.1371/journal.pone.0191442

**Published:** 2018-01-30

**Authors:** Cornelis P. L. Paul, Theodoor H. Smit, Magda de Graaf, Roderick M. Holewijn, Arno Bisschop, Peter M. van de Ven, Margriet G. Mullender, Marco N. Helder, Gustav J. Strijkers

**Affiliations:** 1 Department of Orthopedic Surgery, Academic Medical Center, University of Amsterdam, Amsterdam, The Netherlands; 2 Department of Medical Biology, Academic Medical Center (AMC), University of Amsterdam, Amsterdam, The Netherlands; 3 Department of Orthopedic Surgery, VU University Medical Center, Amsterdam Movement Sciences, Amsterdam, The Netherlands; 4 Department of Epidemiology and Biostatistics, VU University Medical Center, Amsterdam, The Netherlands; 5 Department of Plastic, Reconstructive and Hand Surgery, VU University Medical Center, Amsterdam, The Netherlands; 6 Department of Oral and Maxillofacial Surgery, VU University Medical Center, Amsterdam, The Netherlands; 7 Department of Biomedical Engineering and Physics, Academic Medical Center (AMC), Amsterdam, the Netherlands; University of Crete, GREECE

## Abstract

**Introduction:**

Low-back pain (LBP) has been correlated to the presence of intervertebral disc (IVD) degeneration on T2-weighted (T2w) MRI. It remains challenging, however, to accurately stage degenerative disc disease (DDD) based on T2w MRI and measurements of IVD height, particularly for early DDD. Several quantitative MRI techniques have been introduced to detect changes in matrix composition signifying early DDD. In this study, we correlated quantitative T2, T1rho and Apparent Diffusion Coefficient (ADC) values to disc mechanical behavior and gold standard early DDD markers in a graded degenerated lumbar IVD caprine model, to assess their potential for early DDD detection.

**Methods:**

Lumbar caprine IVDs were injected with either 0.25 U/ml or 0.5 U/ml Chondroïtinase ABC (Cabc) to trigger early DDD-like degeneration. Injection with phosphate-buffered saline (PBS) served as control. IVDs were cultured in a bioreactor for 20 days under axial physiological loading. High-resolution 9.4 T MR images were obtained prior to intervention and after culture. Quantitative MR results were correlated to recovery behavior, histological degeneration grading, and the content of glycosaminoglycans (GAGs) and water.

**Results:**

Cabc-injected IVDs showed aberrancies in biomechanics and loss of GAGs without changes in water-content. All MR sequences detected changes in matrix composition, with T1rho showing largest changes pre-to-post in the nucleus, and significantly more than T2 and ADC. Histologically, degeneration due to Cabc injection was mild. T1rho nucleus values correlated strongest with altered biomechanics, histological degeneration score, and loss of GAGs.

**Conclusions:**

T2- and T1rho quantitative MR-mapping detected early DDD changes. T1rho nucleus values correlated better than T2 and ADC with biomechanical, histological, and GAG changes. Clinical implementation of quantitative MRI, T1rho particularly, could aid in distinguishing DDD more reliably at an earlier stage in the degenerative process.

## Introduction

Low-back pain (LBP) is a major medical problem and one of the most frequent reasons for people to seek medical care in Western societies [1, 2]. Multiple large general population-based studies in the last decade have provided strong evidence that changes in magnetic resonance imaging (MRI) appearance of the lumbar intervertebral discs (IVD) correlate to degeneration and LBP symptoms [3–5] and may aid in the assessment of severity of degenerative disc disease (DDD) [6]. Moreover, it has been shown that in teenagers and young adolescents, early symptomatic DDD is prognostic for exacerbation of LBP complaints and progression of DDD in adulthood [7–11]. This leads to school- and work absenteeism, disability and/or medical intervention, all adding to the enormous socio-economic costs of LBP [1, 12, 13].

At the onset of the degenerative cascade of DDD, the nucleus pulposus (NP) of the disc loses proteoglycans (PGs), decreasing the discs’ capability to imbibe and retain water [14]. This is thought to be the initial step in the vicious degenerative cycle: the PG drop detrimentally affects the IVDs biomechanical properties, hampering the discs ability to recover from daily loading conditions, causing cumulative loss of IVD height and making the disc prone to further degradation [15].

Reliable detection of early DDD is challenging. Currently, conventional T1w-, T2w- and PD-MR imaging are successfully employed in the clinical work-up of low-back pain patients with suspected (advanced) DDD [16]. When IVD height and T2-weighted (T2w) signals are lower than adjacent counterparts and/or what can be expected from natural ageing, degenerative disc disease (DDD) is considered as a possible causal factor for the LBP [17–19]. Inherently, this is at a point where the disc has already suffered an MRI detectable amount of matrix degradation and height loss, and therefore the changes in T2 signal are apparent only at on advanced stage of DDD. An additional drawback is that MRI provides only limited specificity on the structural origin of the signal intensity changes [20, 21]. A decrease in T2w signal is generally attributed to a decrease in water content, but the source of signal intensity changes cannot be specified. Furthermore, it is difficult to determine whether water loss is due to daily activity and circadian (diurnal) rhythm (which is known to influence both water and height parameters) [22], or a water content drop in the ECM due to a loss of proteoglycans (PG’s) from the matrix and therewith the inability to retain water [23–25]. Finding matrix degeneration on T2w-images can also be obscured or mimicked by influx of fluids during an infectious or inflammatory process [26] or by the presence of free water in the lacunas of degraded ECM [27]. In addition, T2w- and PD-imaging are not informative of the discs’ mechanical (dys)function, or the cause of degeneration and pain [28–32]. Taken together, it is clear that additional non-invasive imaging methods are desired to address early onset DDD [7, 19, 33–36].

New MRI techniques sensitive to proton-matrix interactions (PG-bound water), matrix-organization, and water diffusion, rather than water-content only, could therefore prove more meaningful in identifying early DDD [7, 33–36]. A promising MRI contrast for imaging the PG-rich nucleus region is T1rho. T1rho is an MRI relaxation time parameter, which is sensitive to low-frequency interactions between macromolecules, such as PGs, and bulk water. T1rho correlates better with PG matrix content in cartilaginous tissue than T2 [27, 37]. Moreover, T1rho displays a higher dynamic (imaging) range than T2, which improves the ability to detect changes in early osteoarthritis [38]. In a study by Nguyen *et al*. T1rho values in the nucleus were reported to correlate strongly with GAG-content and mechanical properties, such as swelling pressure [39].

The annulus fibrosus (AF) is a highly-organized tissue with a far lower PG content than the nucleus. In healthy discs its porous structure aids fluid-exchange of the nucleus. This structural organization and porosity is lost with degeneration [40]. Diffusion-tensor imaging (DTI) provides another promising MRI contrast for diagnosis of early onset DDD. DTI enables quantification of the water apparent diffusion constant (ADC) in tissue, which strongly depends on the structural integrity of the tissue [41, 42]. Recent reports on DTI of the IVD show that ADC can distinct diurnal fluid flow changes [42] and regional variations (endplate to annulus) of fluid flow changes in IVD degeneration [43, 44]. Furthermore, a study from Manac'h et al. (2013) shows that ADC was the only MR sequence able to detect change during compression of IVDs due to fluid-flow direction shifts [25].

The aim of this study was to assess whether early onset DDD can be detected using quantitative T1rho and ADC mapping and compare results to T2-mapping. To this end, have used a high-resolution 9T MRI scanner and an established *ex vivo* IVD degeneration model. Chondroïtinase ABC (Cabc) injection in the nucleus was used to selectively induce a small GAG loss, to mimic the primary phase of disc degeneration [45–47]. IVDs were cultured in a bioreactor for 20 days, this standardizes culture environment (pH stable, osmotic conditioned culture medium) and loading conditions (with continuous monitoring) to ensure comparability between experimental groups [48–50]. We correlated T1rho and ADC mapping to accepted parameters for IVD degeneration, including histological score, biochemical content (water and GAG), and biomechanical parameters. Correlations were compared to conventional quantitative T2-mapping.

## Material & methods

### IVD specimens

**Fig 1:**
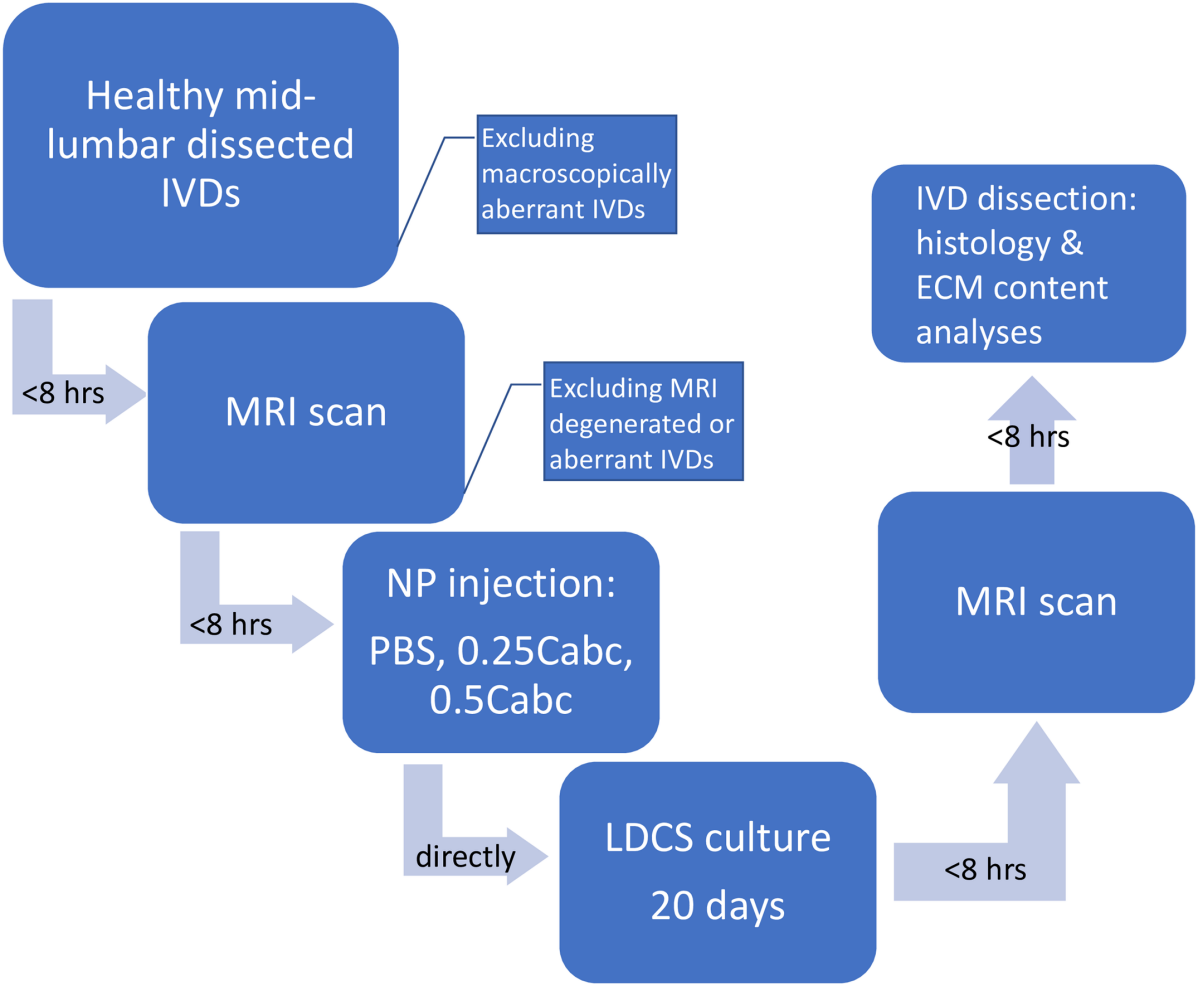
Process map showing the IVD specimen handling steps after dissection of the lumbar caprine IVDs from the spine.

Cadaveric lumbar spines (n = 12) from healthy skeletally mature female (3–5 year-old) slaughter goats (Capra aegagrus hircus, sub breed Dutch white milk goat) were obtained from an abattoir. The lumbar spine and IVD of this specific goat species has been extensively studied and shown to closely resemble the human IVD with respect to biomechanical properties, cell population (no notochordal cells) and matrix composition (18;21;39–41). Cadaver caprine spines used in the current study were obtained after slaughter from an abattoir in The Netherlands (Firma vd Horst, Maarssen, N 52° 09.008' E 005° 01.327'). As we use remnants of slaughter animals no approval of an ethical committee is required. Within 3 hours after slaughter, the exterior of the lumbar spines was sterilized using a medical grade iodide-alcohol solution prior to dissection under sterile conditions of the lumbar IVDs. IVDs with adjacent cartilaginous endplates were dissected using an oscillating surgical saw. The discs were dissected by sawing in two parallel planes as close as possible to the proximal and distal endplates, preserving the cartilaginous endplate but removing any excess bone tissue. The sawing planes were perpendicular to the central axis of the individual motion segment. IVDs were cleaned with sterile gauze to remove any debris, blood and muscle or ligament tissue (especially remainders of the posterior longitudinal ligament). IVDs were inspected for signs of degeneration (hernia’s, osteophytes, periarticular sclerosis, fused motion-segments) or anomalies. If an IVD had any degenerative changes or aberrancies it was excluded. Mid-lumbar disc levels L2-L3, L3-L4 and L4-L5 (n = 24 total) were used in the culture experiments, as these disc levels do not significantly differ in height, size, mechanical properties or matrix content [46, 49–51]. Still, all experimental groups had an equal distribution of disc levels. IVDs were then placed in a 6-wells plate with culture medium prior to MRI scanning and placement in the bioreactor. IVDs with anomalies on the pre-experimental MRI were also excluded. After the pre-scan, IVDs were randomly assigned to one of 3 experimental groups: 1. an injection control group injected with 100uL of Phosphate Buffered Saline solution (PBS 10x; n = 8); 2. a Chondroïtinase ABC (Cabc) group injected with 100uL of 0.25u/ml Cabc solution (n = 8); or 3. a Chondroïtinase ABC (Cabc) group injected with 100uL of 0.5u/ml Cabc solution (n = 8). The injection method was standardized; both groups were punctured via the left lateral annulus with a 32G needle and the solution was injected slowly into the nucleus, prior to placement in the culture chamber (Fig 1).

### IVD culture and loading

IVDs were cultured for 20 days in individual culture chambers in the previously described Loaded Disc Culture System (LDCS) (39), which is housed in an incubator at 37°C, 95% humidity, and 5% CO2. Discs were cultured in standard DMEM (Gibco, Paisley, UK) with 10% FBS (HyClone, Logan, UT), 4.5g/L glucose (Merck, Darmstadt, Germany), 50 μg/ml ascorbate-2-phosphate (Sigma Aldrich, St. Louis, MO), 25 mmol/L HEPES buffer (Invitrogen), 10,000 u/ml penicillin, 250 μg/L streptomycin, 50 μgr/mL gentamicin and 1.5 μgr/mL amphoterizin B (all from Gibco). These culture conditions were previously optimized to maintain the IVDs native properties in a bioreactor for up to 21 days [50, 52–54]. Medium (40 ml per culture chamber) circulated continuously (3ml/h) using a peristaltic pump and was exchanged every 48 hours and checked for pH (7.2–7.4) and osmolarity (360–380 mOsm; measured by cryoscopy).

**Fig 2:**
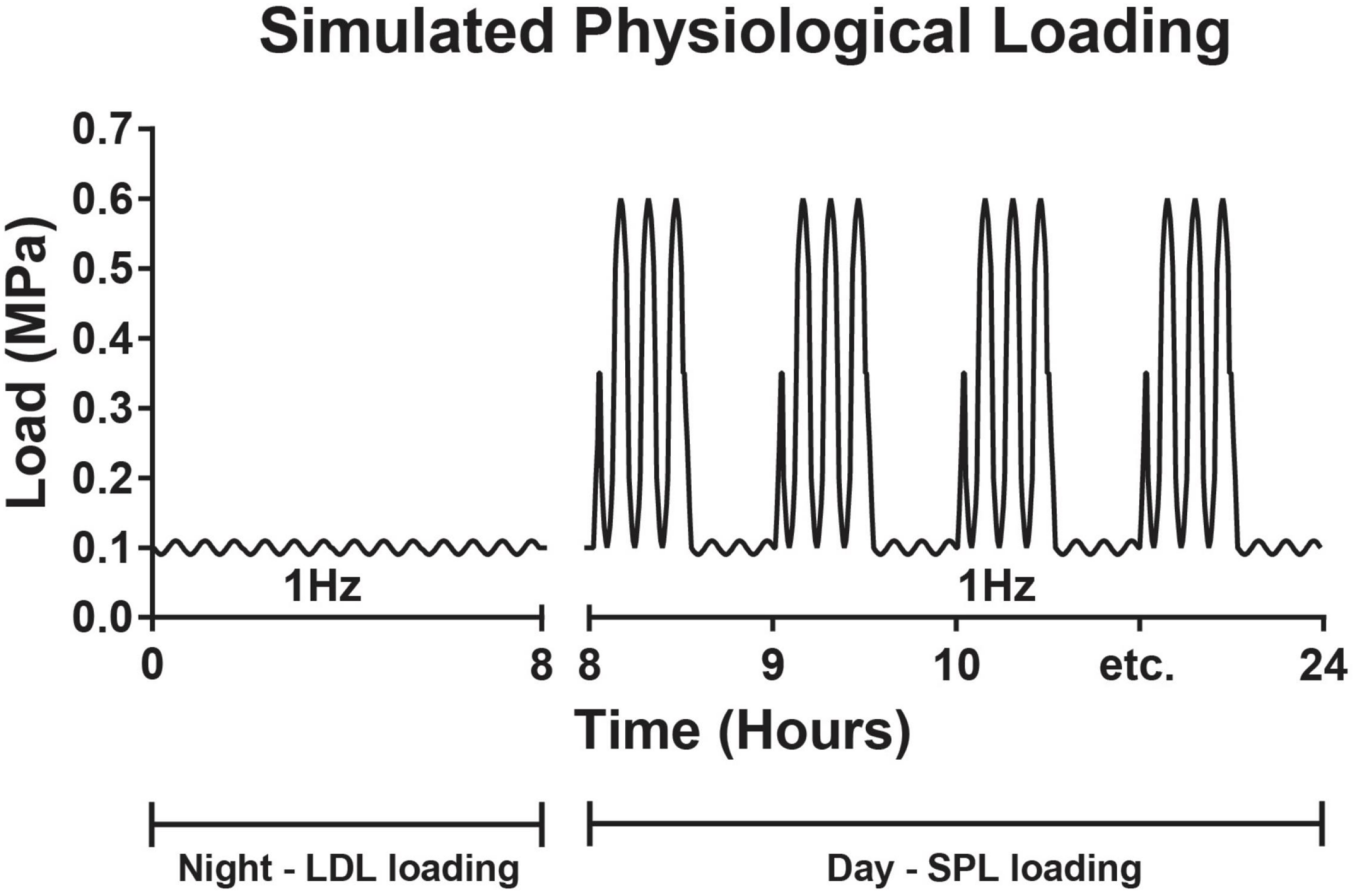
Scheme of the daily simulated-physiological loading (SPL) regimes. On the Y-axis the axial load (MPa) as applied on the IVDs. All IVDs started with 8 hours of low dynamic load (LDL) around 0.1 MPa, after which a 16 hour SPL loading regime was applied as indicated in the caption, this diurnal regime is repeated daily.

During culture, IVDs were loaded with dynamic axial loading. Loading magnitudes and frequency were derived from *in vivo* pressure measurements in a lumbar segment of a goat during different activities (*e*.*g*. lying down, walking and jumping on a haystack) [55]. Firstly, all discs were subjected to a low sinusoidal load (Low Dynamic Load, LDL; 0.09–0.11 MPa; 1Hz) during the first 8 hours of culture to investigate the response of the disc to initial low axial loading: if an IVD showed large aberrancies in its baseline subsidence behavior, it was excluded. However, in the current study this did not occur. All IVDs were subsequently subjected to a simulated-physiological load (SPL), consisting of a sinusoidal load (1 Hz) alternating in magnitude every 30 minutes (between 0.09–0.11 MPa and 0.1–0.6 MPa) for 16 hours per day, followed by 8 hours of LDL (Fig 2). In previous reports, we have shown that native caprine disc mechanical properties and cell viability can be maintained over 21 days in LDCS culture, when IVDs are loaded with this simulated-physiological loading [49, 50].

### Mechanical data collection

Loading forces during culture were continuously measured with a Kam-e load cell (Bienfait, Haarlem, The Netherlands); disc height changes were measured with an OADM12 opto-electric sensor (Baumer, Berlin, Germany). Both signals were digitized at 100 Hz. We used customized programs in Matlab (Mathworks, Natick, MA, USA) to analyze the data.

To compare the recovery behavior between experimental groups, we quantified parameters of the displacement relaxation curves during the rest phases. For this we fitted a stretched exponential function to the curves. This allows us to characterize the behavior by three descriptive parameters. Displacement relaxation curves during the rest-load sequence (8 hours of LDL) at t = 481 hours (day 20) were used to quantify the effects of injection on IVD biomechanical behavior and compare between groups. We fitted a stretched exponential function to these displacement curves described by [56–58]:

$$\delta =(\delta_{\infty }-\delta_{0})\left[1-e^{\left(-{\left(t/\tau \right)}^{\beta }\right)}\right]$$ (1)

In this function *δ* (delta) is the displacement of the IVD at time (*t*); *τ* (tau) is the time constant of the function and *β* (beta) is a stretch constant; *δ*_∞_ (delta infinite) is the displacement at *t* = infinite; *δ*_0_ (delta 0) is the displacement at the onset of the curve *t* = 0; *τ* (tau) represents the time required to reach 63% of the asymptotic value after the onset of the loading phase. Parameter *β* modulates the deviation from the standard exponential function described by tau, such that for *β* < 1, deformation is faster than exponential for *t* < tau. When the stretch constant *β* becomes increasingly smaller than 1 and closer to zero, this indicates that a single time constant (tau) was insufficient to describe viscoelastic behaviors and suggests creep behavior is involved due to deformation mechanisms (*e*.*g*. disc bulge and degenerative changes, fluid transport and flow-independent deformations) [56–59]. The LDCS displacement data at *t* = 0 and *δ*_0_ in the stretched exponential were set at 0 for standardization.

### Magnetic resonance imaging (MRI)

MRI scans were performed with a 9.4 T Bruker BioSpec horizontal-bore scanner equipped with an Avance III console and Paravision 5.1 software (Bruker, Ettlingen, Germany). Directly after dissection and inspection (within 8 hours of injection and culture), intervertebral discs were put in a standard 50 mL plastic syringe for the pre-experimental scan. Directly after culture, IVDs were again placed in a 50 mL syringe for the post-experimental scan (within 8 hours after culture). For susceptibility matching and to prevent dehydration of the discs during the MRI measurements, the syringe was filled with culture medium. The syringe with discs was fixed horizontally in a 35-mm-diameter quadrature send-and-receive birdcage coil (Bruker) and placed in the MRI scanner. The central axis of the disc was aligned with the B0 magnetic field of the MRI scanner, which means that the ligament fibers of the annulus fibrosis were all aligned at approximately the same angle with B0, minimizing the orientational dependence of the relaxation times caused by dipolar interactions of fiber-bound water molecules.

The MRI protocol comprised of 2D multi-slice T2-weighted (T2w) imaging, T2-mapping, T1rho-mapping, and diffusion-tensor-imaging (DTI) for ADC-mapping. Unless stated otherwise, orientation = axial, number of slices = 9, slice thickness = 1 mm, and field-of-view (FOV) = 30 x 30 mm^2^. Other acquisition parameters were as follows. T2w: sequence = RARE, repetition time (TR) = 2500 ms, echo time (TE) = 36 ms, RARE-factor = 8, number of averages (NA) = 8, encoding matrix = 384 x 384, reconstruction matrix = 384 x 384, acquisition time = 16m0s. T2-mapping: sequence = multi spin-echo, TR = 3000 ms, TE = 9, 18, 27, 36, 45, 54, 63, 72, 81, 90, 99, and 108 ms, NA = 2, encoding matrix = 128 x 96, reconstruction matrix = 128 x 128, acquisition time = 9m36s. T1rho-mapping: sequence = spin-lock preparation with gradient-echo readout, number of slices = 1, TR = 5000 ms, TE = 5 ms, spin-lock RF amplitude = 1000 Hz, spin-lock time (SLT) = 5, 10, 20, 40, 60, 80, and 100 ms, NA = 1, encoding matrix = 64 x 64, reconstruction matrix = 128 x 128, acquisition time = 37m20s. DTI: sequence = spin-echo with Stejskal-Tanner diffusion gradients, TR = 2000 ms, TE = 20 ms, number of diffusion gradient directions = 10, diffusion b-value = 1000 s/mm^2^, one image with b-value = 0 s/mm^2^, NA = 1, encoding matrix = 128 x 64, reconstruction matrix = 128 x 128, acquisition time = 23m28s. The T1rho pulse sequence was performed with a spin-lock preparation compensated for B1 and B0 field imperfections [60].

### IVD region definition and image analysis

**Fig 3:**
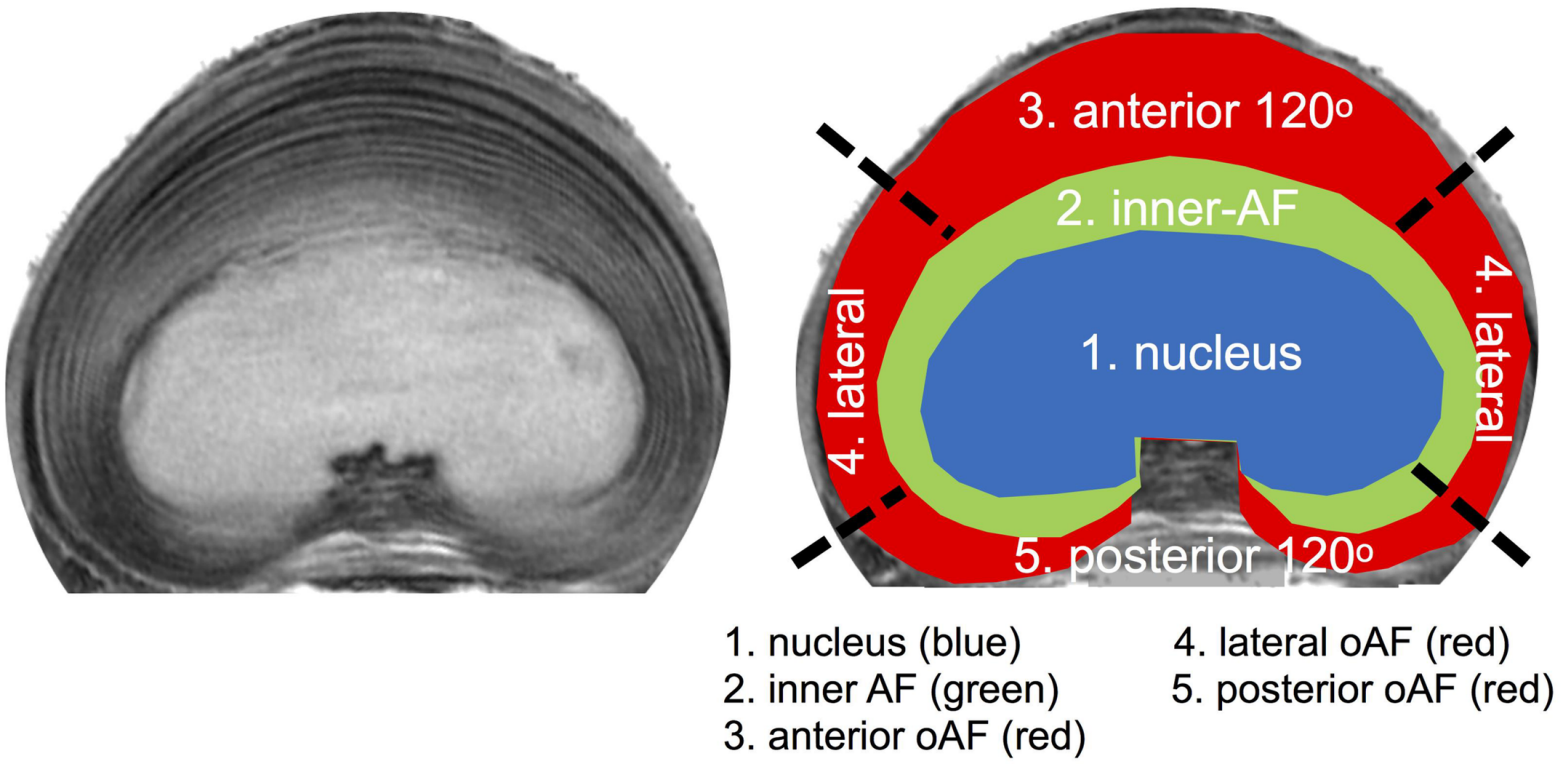
IVD Region definition. High resolution T2w MRI of a mid-height transverse slice (left) with a schematic representation of the five ROIs drawn in the image on the right. The nucleus, inner- and outer-annulus region were separated by their signal intensity and morphological appearance on the transverse image. The anterior and posterior outer-annulus were defined as the most anterior and posterior 120o of outer-annulus laminae. The lateral section comprised the 60o sections in between anterior and posterior outer-annulus on each side. The mean signal intensity of the three outer-annulus regions was calculated from the signal intensity measured in two separate areas on opposite sides of the IVD. The posterior notch was omitted for analyzes because of lack of signal homogeneity.

Calculation of the parametric maps was performed in Mathematica (Wolfram Research, Inc.). Quantitative T2 values were calculated by pixel-wise fitting of signal intensities S to the equation S(TE) = S_0_ e^-TE/T2^. T1rho values from fits to the equation S(SLT) = S_0_ e^-SLT/T1rho^. The apparent diffusion coefficient (ADC) was calculated using the Bruker Paravision 5.1 software. Subsequently, the central slice of the stack of 9 slices through the middle of the intervertebral disc was used for further evaluation. A region of interest (ROI) outlining the nucleus pulposus was manually drawn in Mathematica (Fig 3). Quantitative parameter values (T2, T1rho, and ADC) are reported as mean ± standard deviation within the ROI. The 3D-T1w images were exported in Dicom format and imported in RadiAnt (Medixant, Poland) Dicom viewing software. The length tool was used to quantify disc height.

### Histology

Directly after culture and MRI scanning, the IVDs were divided in halves; the left half was used for histological sectioning and the right half was used for matrix water and GAG content measurements. Histology halves were fixed in formaldehyde and decalcified using Kristensen's solution (formic acid decalcifier buffered with formate). A midsagittal tissue slice of 1 mm was cut with a scalpel and embedded separately in paraffin for sagittal sectioning. The rest of the IVD was embedded and cut for transverse sectioning. Three 3-μm thick sections were cut using a standard microtome and subsequently stained with a standard H&E, safranin-O, and alcian-blue. Histological classification of degeneration was performed according to the Rutges score. This is a descriptive histological score that quantifies degenerative changes in the NP, AF and endplate using both transverse and sagittal sections. On a continuous scale, 0–2 signifies a healthy non-degenerated disc, 3–5 mild degeneration, 6–8 moderate degeneration, 9–11 severe degeneration, and a score of 12–15 being completely degenerated [46, 61].

### Water and GAG measurement

Quantitative biochemistry was performed after culture on tissue samples taken from nucleus, inner annulus (iAF), and outer annulus (oAF) regions of the IVDs (n = 8 for each region). Water content of each sample was calculated from measured wet (ww) and dry weights (dw), before and after freeze drying (speedvac). Dry weight samples (~1 mg) were digested in a papain-digestion (5 mmol/L L-cysteine, 50 mmol/L EDTA, 0.1 M sodium acetate, pH titrated to 5.53 using 1 M NaOH and 3% (v/v) papain) at 65uC. Papain-digestion suspension (10 mL) was analyzed using a 1.9-dimethyl-methylene blue (DMMB) assay (Biocolor Ltd., Carrickfergus, UK) in accordance with the manufacturer’s description and measuring light absorption of samples with a spectrophotometer at a wavelength of 656 nm. Measured amount of glycosaminoglycans (GAG) for each sample was normalized to tissue dry weight.

### Statistical analysis

Descriptive data are presented as means ± standard deviation (SD). Effects of intervention on MRI quantitative values, day 20 stretch-exponential parameters and post-experimental mean percentage water and GAG content were tested using Generalized Estimating Equations (GEEs). An exchangeable correlation structure was used to take into account correlation between outcome parameters observed on IVDs of the same goat. Robust standard errors were used to take into account possible misspecification of the correlation structure. The models contained fixed effects for intervention (PBS, 0.25 Cabc and 0.5 Cabc) and/or fixed effects for ROI (when comparing means between nucleus and inner-annulus and means within the outer-annulus regions). Similarly GEE analysis was used to compare the mean percentage change in T2 and T1rho. For this comparison the fixed effect was an indicator for T2% change versus T1rho % change and an exchangeable correlation structure was used to account for correlation within IVD. In case of a significant effect of intervention or ROI, posthoc tests were compared with a Bonferroni correction in order to decide which pairs of interventions or ROIs differed significantly in terms of their means. William’s test was used for comparison of dependent correlations (*e*.*g*. correlation between %water and T2 post-experiment and correlation between %water and T1rho post-experiment). GEE analyzes were performed in SPSS 24 (IBM Corporation, Armonk, NY, USA). William’s test was performed in R 3.2.5. version using the psych package (The R Foundation for Statistical Computing, 2016). P-values below 0.05 were considered significant.

## Results

Baseline values of the pre-experiment scans are shown in Table 1 for all three MR sequences per disc region and per lumbar level. There were no significant differences in values of regions between scanned level. For T2, T1rho and ADC mean regional values differed significantly between the NP and iAF (T2 and T1rho *p* < 0.001; ADC *p* = 0.02). For T2 and T1rho the separate oAF regions also differed significantly (all *p* < 0.001). For ADC the separate outer-annulus values did not differ significantly. Between experimental groups, discs from the three lumbar levels were equally distributed (three L2-L3 and L3-L4 discs, and two L4-L5 discs) and no significant differences in pre-experimental (baseline) scan values were found (mean ± standard deviation MRI values of pre- and post-experimental T2, T1rho, and ADC scans per disc region are shown in S1 Table with all pre- and post-experimental MRI values per disc region).

**Table 1:** Baseline T2, T1rho, and ADC values (mean ± SD) per ROI and disc level.


ROI	Level	T2 (ms)	T1rho (ms)	ADC (x10^-3^ mm^2^/s)
NP	L2-L3	65.8 ± 10.3	133.2 ± 24.2	1.2 ± 0.2
	L3-L4	61.6 ± 11.7	122.6 ± 42.3	1.3 ± 0.2
	L4-L5	63.4 ± 10.7	130.1 ± 25.3	1.2 ± 0.2
iAF	L2-L3	40.2 ± 4.9	74.8 ± 7.2	1.2 ± 0.1
	L3-L4	40.6 ± 7.2	75.1 ± 8.2	1.1 ± 0.2
	L4-L5	40.2 ± 4.7	73.8 ± 6.1	1.2 ± 0.1
oAFa	L2-L3	26.0 ± 4.1	36.0 ± 5.4	1.1 ± 0.4
	L3-L4	26.7 ± 2.7	37.3 ± 3.8	1.0 ± 0.2
	L4-L5	24.2 ± 5.4	34.8 ± 3.0	1.0 ± 0.4
oAFl	L2-L3	30.6 ± 5.4	49.0 ± 12.0	1.2 ± 0.3
	L3-L4	27.5 ± 5.7	44.0 ± 6.0	1.0 ± 0.1
	L4-L5	30.3 ± 3.1	44.5 ± 6.9	1.1 ± 0.3
oAFp	L2-L3	27.8 ± 3.1	40.8 ± 5.7	1.1 ± 0.3
	L3-L4	26.3 ± 2.9	39.2 ± 3.5	1.0 ± 0.2
	L4-L5	27.2 ± 3.2	38.5 ± 4.5	1.0 ± 0.3

### MRI pre- and post-experimental scan values

**Fig 4:**
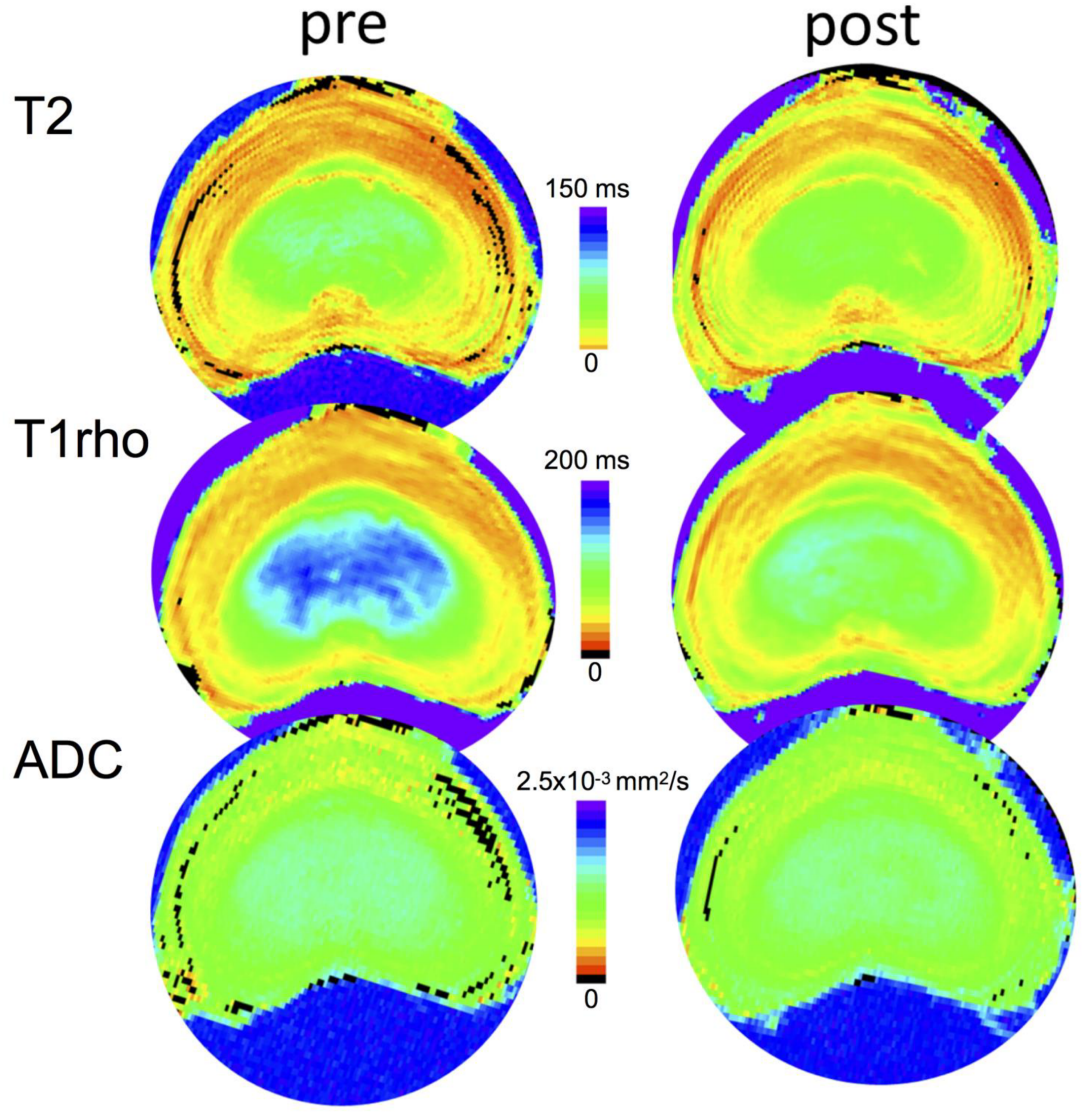
Representative quantitative parameter maps (T2, T1rho, and ADC) of a mid-level transverse slice of 0.5 Cabc-injected IVD pre- and post-experiment. Color scales are 0-150ms, 0-200ms, and 0–2.5x10-3 mm2/s, for T2, T1rho, and ADC, respectively.

Fig 4 shows representative images of pre- and post-experiment maps (top to bottom; T2, T1rho, and ADC) of a 0.5Cabc injected IVD.

**Fig 5:**
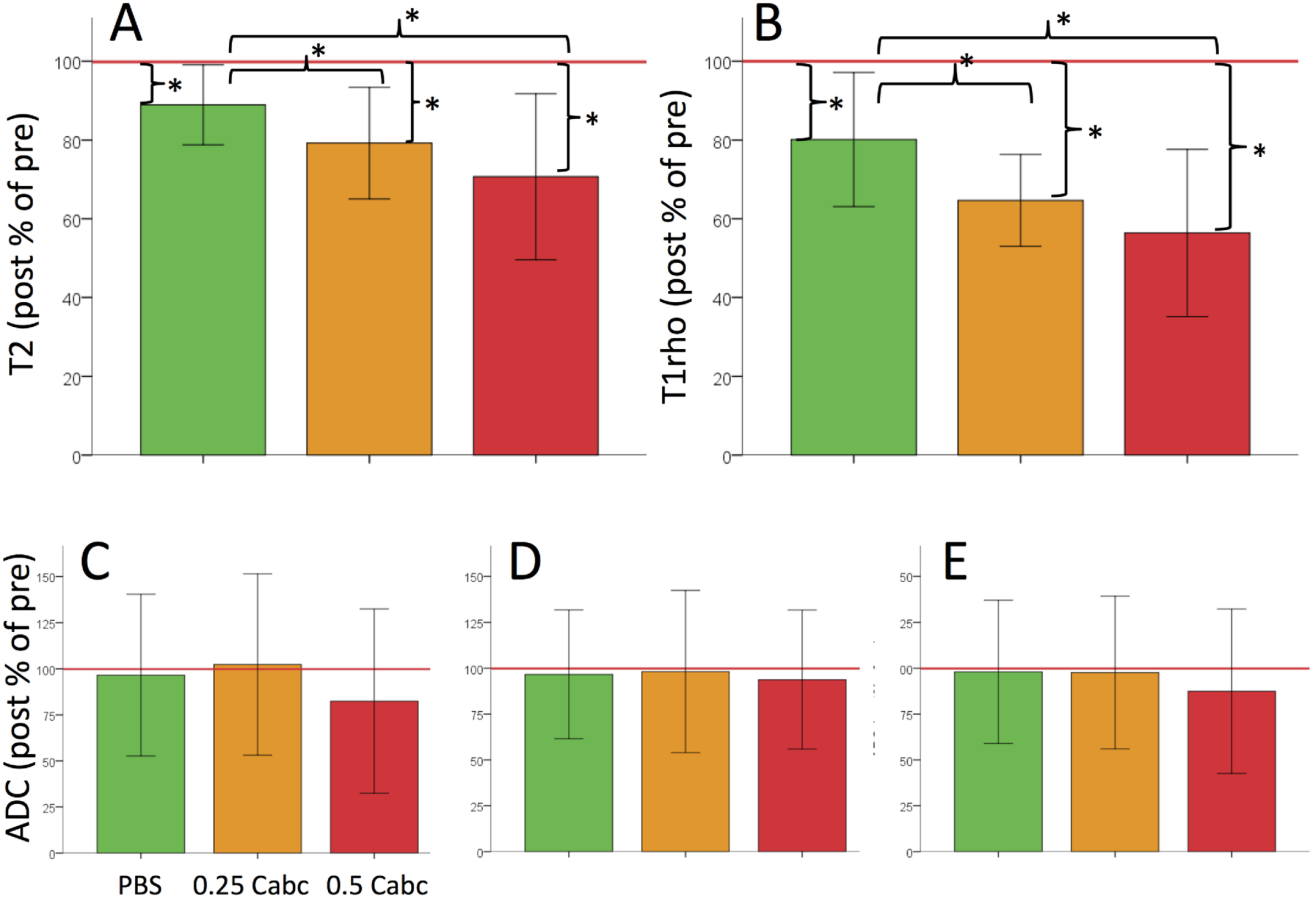
Bar graphs showing mean ± SD post-to-pre differences (mean difference of sample paired post-experiment value to pre-experiment differences) for all three experimental groups. Results are shown of the T2 (a) and T1rho (b) values in the nucleus and ADC in the outer-annulus; anterior (c), lateral (d) and posterior (e). PBS clearly had the least influence on quantitative MRI values for all three parameters, whereas 0.25 and 0.5 Cabc show larger loss of signal in the nucleus. Asterisk (*) indicates p-values below 0.05.

The difference in paired pre- and post-experimental scan values per sample are expressed as mean % loss (post to pre) in Fig 5. Between the experimental groups the pre-to-post difference in T2 and T1rho are larger (differ more from 100%) for the Cabc injections in general and for the higher dose in particular. The signal loss post to pre was significant (*p* < 0.001) in all three experimental groups in the nucleus region for T2 and T1rho (Fig 5). When comparing signal losses between experimental groups, T2 signal loss in the nucleus was significantly larger in the 0.25 Cabc group (*p* = 0.04) and the 0.5Cabc group (*p* < 0.001) when compared to changes in the PBS group. T1rho post-experiment signal decrease was also significantly larger in both the 0.25 Cabc (*p* = 0.001) and the 0.5 Cabc (*p* < 0.001) group when compared to changes in the PBS group (Fig 5A and 5B). T2 and T1rho changes for the inner- and outer annulus regions in the Cabc groups did not differ significantly when compared to the PBS control group. No ADC signal change was significantly different in any region when compared to PBS (Fig 5C, 5D and 5E).

### Correlation of MRI with recovery behavior

Changes in recovery behavior due to injection of PBS or Cabc are shown in Table 2. As expected, Cabc injection had a dose-related effect on the IVD’s poro-elastic behavior. The tau (time-constant) increases significantly more compared to PBS in the 0.25 Cabc (*p* = 0.024) and 0.5 Cabc (*p* < 0.001), and 0.5 more than 0.25 Cabc (*p* = 0.005). The beta (stretch-constant) decreased significantly in the 0.5 Cabc group when compared to PBS (*p* = 0.006). The delta infinite (height loss) also changed significantly in the 0.5 Cabc groups when compared to PBS (*p* < 0.001) and 0.25 Cabc (*p* = 0.02).

**Table 2:** Stretched exponential fit parameters for the recovery curves at day 20.


Exp. group	τ (tau)	β (beta)	δ_∞_ (delta infinite)
PBS	3.08 ± 0.61	1.01 ± 0.12	-4.6 ± 0.8
0.25 Cabc	3.74 ± 0.62	0.87 ± 0.14	-5.2 ± 0.9
0.5 Cabc	5.54 ± 1.41	0.70 ± 0.24	-6.4 ± 1.2

Descriptive parameters (mean ± SD) from the stretched-exponential fits of the recovery deformation curves during the last LDL loading at day 20 for all three experimental groups.

**Fig 6:**
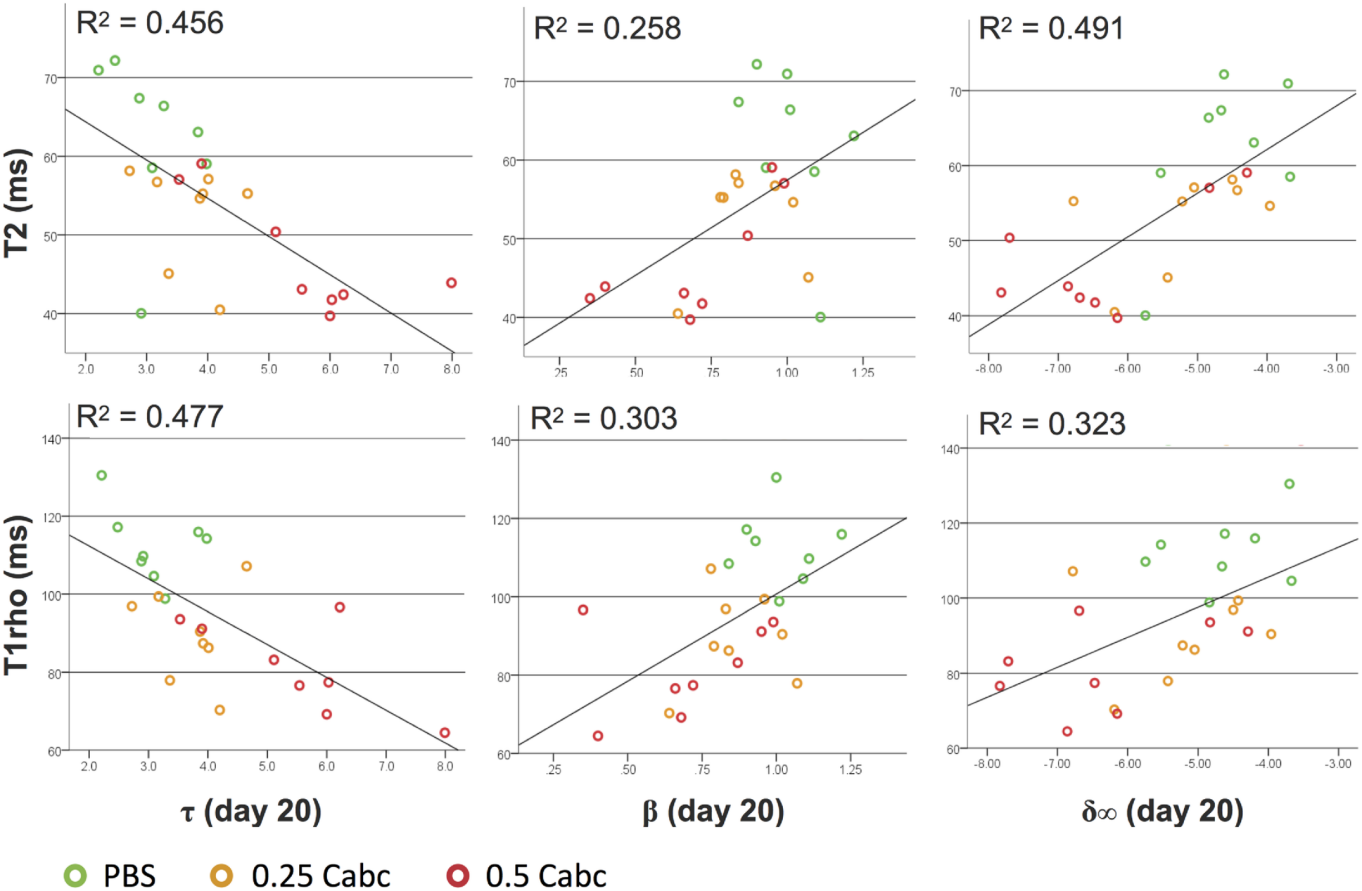
Correlation of post-experiment T2 (top row) and T1rho (bottom row) nucleus values with day 20 stretched-exponential parameters tau, beta and delta infinite.

The measures of disc biomechanics during recovery at day 20 are correlated to the post-experimental MR scan values (Fig 6). T2 and T1rho nucleus values have an equal negative moderate correlation with tau (r = -0.675 and r = -0.691, respectively; not significantly different). For beta both have a moderate positive correlation.

**Fig 7:**
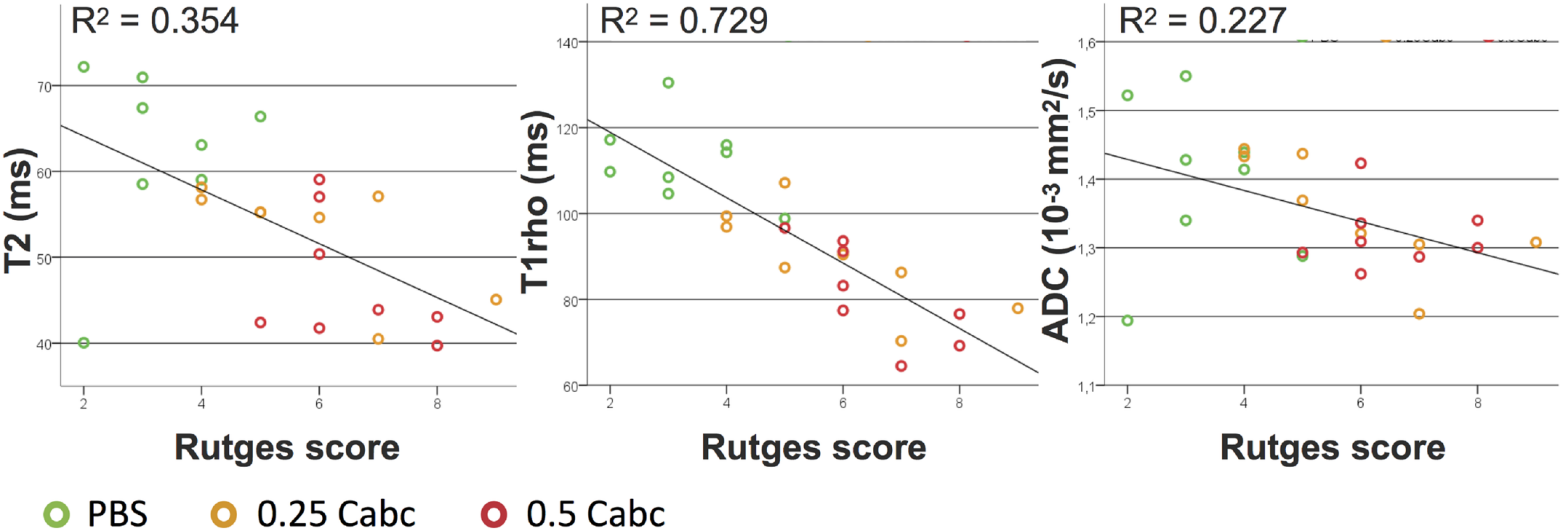
Correlation of post-experiment MR T2, T1rho, and ADC nucleus values with Rutges histological degeneration score.

In Fig 7 the correlations are shown between the T2, T1rho and ADC post-experiment nucleus measurements and the histological score for degeneration. The Rutges score in the PBS group was the lowest; 2 to 5 (mean score = 3,6), corresponding with no or mild degenerative changes. Both Cabc groups had scores ranging between 4 and 9 (0.25 Cabc mean score = 5.8; 0.5 Cabc mean score = 6.2), showing mild to moderate degenerative changes. T1rho values correlated strongly with the degeneration score (r = -0.854), significantly stronger than T2 and ADC nucleus values (*p* = 0.005 for T1rho compared to T2 and *p* < 0.001 for T1rho compared to ADC).

**Fig 8:**
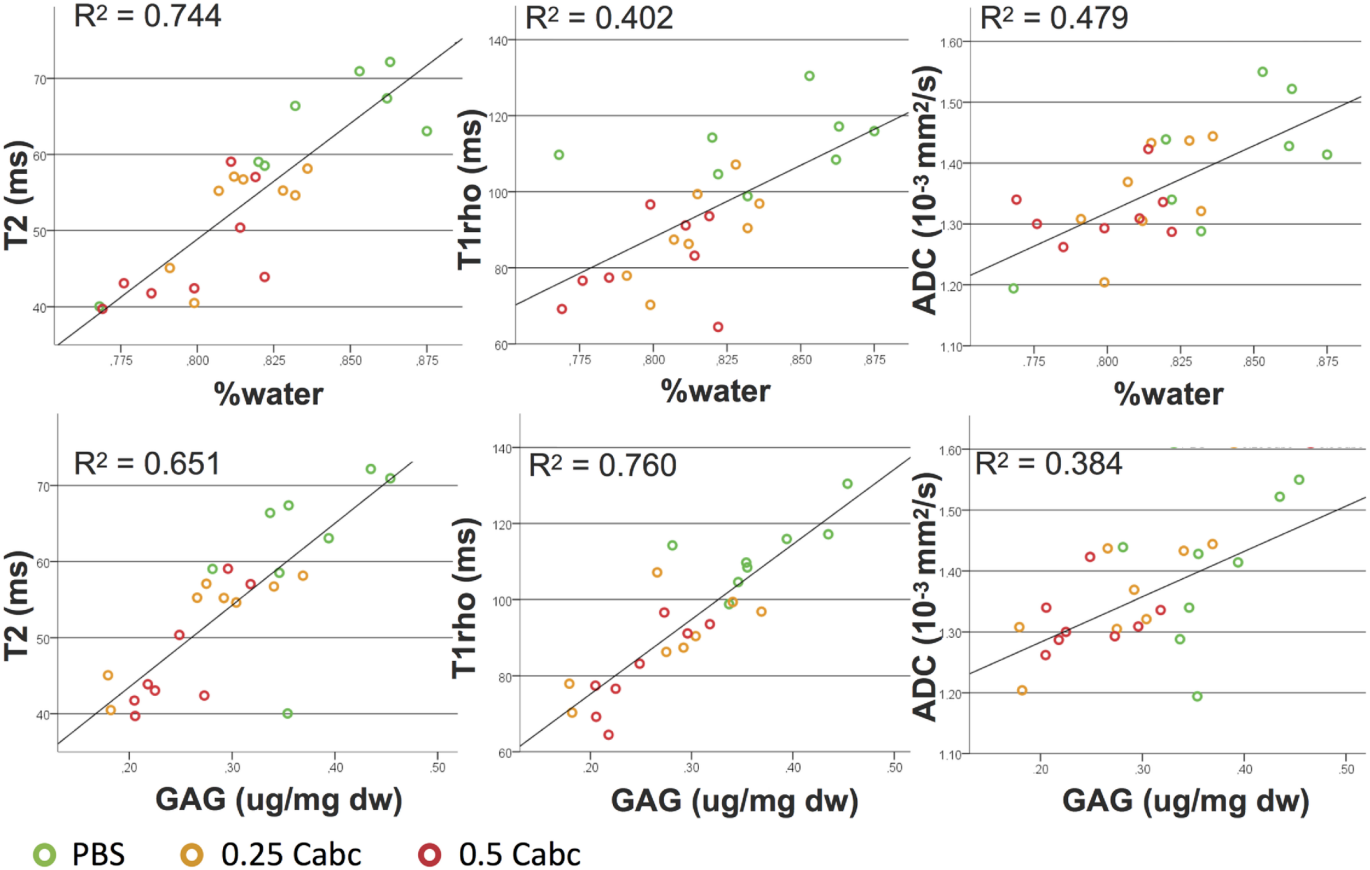
Correlation of post-experiment MR T2, T1rho, and ADC nucleus values to GAG and water content.

The relation between matrix-content in the nucleus (water and GAG) as measured after the experiment and the post-experiment MR values are shown in Fig 8. T2 had a strong positive correlation with water-content (r = 0.863), significantly stronger than T1rho (r = 0.634; *p* = 0.01) and ADC (r = 0.692; *p* = 0.047) (Fig 7). T1rho showed the strongest positive correlation with GAG-content (r = 0.872), significantly stronger than T2 (r = 0.807; *p* = 0.03) and ADC (r = 0.620; *p* = 0.01).

## Discussion

In the current study, we correlated quantitative T2, T1rho and ADC values to disc mechanical behavior, IVD histology and water- and GAG content, to assess their potential for early DDD detection. We demonstrated in our IVD model that quantitative MR mapping with T2 and T1rho, is sensitive to small degenerative changes in the IVDs matrix. To our knowledge, this is the first study to directly compare the correlation of quantitative high-resolution T2, T1rho, and ADC maps with actual disc recovery behavior.

With the use of a 9.4T MRI we were able to image the lumbar caprine IVD in high anatomical detail. We quantitatively mapped the IVD’s 5 distinct regions on T2, T1rho, and ADC images and found significant differences between the nucleus, inner-annulus, and the anterior, lateral and posterior outer-annulus. These regions have previously been described to be distinct on histological appearance, as well as their biochemical composition of the matrix in both human and caprine IVDs [46, 49, 50, 62–65]. The current baseline MR values found for these IVD regions closely resemble values found in similar regional measurements of human lumbar discs [66–70]. Furthermore, baseline values correspond to those found for Pfirrmann grade 1–2 (healthy) human lumbar IVDs, while post-experiment data is similar to grade 2–4 mild degenerative IVDs [67, 69, 71, 72]. This further validates our Cabc-induced degeneration model in lumbar caprine discs as a comparative model for early human IVD degeneration.

T1rho values pre- and post-experiment showed a larger range of values than T2. This is in part due to T1rho having a larger dynamic range than T2 [73–76]. However, the differences found in the correlation of T2 and T1rho and disc mechanical properties, as expressed by the stretched-exponential parameters, show that T1rho values are also more closely linked to actual disc function. Both T2 and T1rho correlated to the Cabc dose-dependent tau increase. T1rho’s higher correlation to beta, is most likely due to T1rho’s stronger correlation to GAG-content. The R-values found in the current study are comparative to T1rho-GAG correlations reported in human [73, 77]. The stretch-constant beta deviates further from 1 (to zero) when poro-elastic properties are lost in favor of more linear (solid-elastic) material behavior [59]. In the case of the IVD, this has been shown to be caused by loss of GAGs (and therewith water) from the NP [78] and structural damage to the disc [57]. Taken together, when lower T1rho values are found, this is representative for the biomechanical deterioration of the poro-elastic properties of the IVD, which is the first step in the degenerative cascade of DDD.

Our data on T2- and T1rho in correlation with recovery behavior are in agreement with numerous other studies. In reports from Mwale *et al*. (bovine tail IVD) and Antoniou *et al*. (human lumbar IVDs) T2 and T1rho values were found to correlate (r-values between 0.6–0.7) with compressive modulus and hydraulic permeability of NP and AF tissue samples of various degenerative states [79, 80]. On human subjects with *in vivo* discography measured “opening pressure” (OP), Borthakur *et al*. reported lower T1rho values and pressures in painful discs with moderate correlation (r = 0.54) of T1rho and OP [81]. Various other reports have shown similar correlations of T1rho to intradiscal pressure [39, 82].

In addition, an important finding in the current study is that in the same IVD samples, T1rho also correlates strongly to the histological degeneration score (r = 0.854) and significantly better than T2 and ADC. The Rutges degeneration score is an adaptation of the traditional Thompson and Boos scores [61]. Besides sagittal and transvers H&E stained sections, it includes a safranin-O and alcian-blue staining of transverse IVD sections. The latter two are both PG-content sensitive staining techniques [83]. Therefore, we feel the found correlation further exemplifies T1rho’s strong affinity with actual GAG-content in the IVDs matrix.

A study limitation can be observed by the loss of T2 and T1rho after culture in all groups, even the PBS injected IVDs. Culture medium is hyperosmotic, but comparable to reported osmotic pressures *in vivo* [53, 54, 84, 85]. This may still have caused slight efflux of water during culture and loading, explaining the overall loss. However, the LDCS culture conditions do not seem influence T2 values greatly, nor the correlation of T2 measures with water-content (r = 0.863), as these are in line with reports in literature for both goat and human lumbar IVDs [86–89].

Another potential limitation of the current study is that the pre- and post-experiment scans were performed while IVDs were unloaded for up to 8 hours and submerged in PBS. All discs are hereby enabled to return to their maximum hydrated and height recovered state [50]. Healthy discs will respond to this environment differently than degenerated discs [52, 54]. This could potentially exacerbate the effects of Cabc-induced degeneration or diminish water-content loss under axial compression in degenerative discs. We observe a slightly higher overall water-content (76–89%) in the IVDs when compared to data from our previous reports (70–85%) [46, 49, 50]. However, we can still quantify T2 and water-content differences between the experimental groups, which refutes the latter. Conversely, the much higher correlation of T2 (r = 0.863) with water-content than T1rho (r = 0.634) could be a resultant of an exacerbating effect of the scanning circumstances (i.e. unloaded and submerged in PBS), although found correlations are consistent with those found in other studies [39, 77, 87, 89–91]. This underlines a very important message, namely that T2 measurements are sensitive to the interaction of degenerative changes, disc environment, and scanning circumstances, whereas T1rho measures –correlating more to GAG content– are not.

The current study does not provide much support for the utility of ADC in detection of early DDD. In part this is due to the limitations of the study design, because we only injected the NP with Cabc. The ADC of the AF can be expected to be most sensitive to degenerative changes, because of its highly structured organization. However, the limited culture period makes degenerative changes in the AF as a result of the injection in the NP improbable. Other study limitations could also attribute to a lack of signal change specifically for the ADC: while scanned the IVDs were unloaded, at room temperature, and submerged. The influence of this static hydrated equilibrium on the ADC signal is unknown. However, others have found no influence of an unloaded or compressive state of IVDs with respect to T1 and T2 [25].

In summary, the current region-specific results and found correlations in our lumbar caprine IVD model, demonstrate that T1rho nucleus values correlate with GAG content, histological degeneration, as well as disc mechanical properties to a higher degree than T2 and ADC. We speculate selective implementation of T1rho, e.g. when (early) DDD is expected but T2 images do not confirm the suspicion, could aid in observing degenerative changes with more certainty in clinical practice [92]. Potentially, with improved patient selection at an early disease stage with the use of optimized MRI techniques, early intervention (using therapeutics such as anti-inflammatory-, disease-modifying- or regenerative drugs) might yield more success in relieving symptoms and preventing progression of the IVDs degenerative cascade.

## Supporting information

S1 Table With all pre- and post-experimental MRI values per disc region. This supplementary table shows all pre- and post-experimental MRI values of T2, T1rho, and ADC maps (mean ± SD) per experimental group and disc region. There were no significant differences in values between experimental groups per ROI. Of note: for the statistical analyses in the study the differences (delta values) between paired pre-post measurements per sample within a group were used (and the mean ± standard deviation of these differences) and not overall experimental group means as inter- and intra-individual variation would obscure intervention effects.
(DOCX)
