# Supplementary material for: Quantitative MRI in early intervertebral disc degeneration: T1rho correlates better than T2 and ADC with biomechanics, histology and matrix content
Source: PLoS One. 2018 Jan 30;13(1):e0191442. doi: 10.1371/journal.pone.0191442 (PMC5790235; doi:10.1371/journal.pone.0191442)
Supplement: S1 Table — This supplementary table shows all pre- and post-experimental MRI values of T2, T1rho, and ADC maps (mean ± SD) per experimental group and disc region. There were no significant differences in values between experimental groups per ROI. Of note: for the statistical analyses in the study the differences (delta values) between paired pre-post measurements per sample within a group were used (and the mean ± standard deviation of these differences) and not overall experimental group means as inter- and intra-individual variation would obscure intervention effects. (DOCX) [file pone.0191442.s001.docx]

S1 Table with all pre- and post-experimental MRI values per disc region

|  | | | | | | | |
| --- | --- | --- | --- | --- | --- | --- | --- |
|  | | T2 (ms) | | T1rho (ms) | | ADC (x10^-3^ mm^2^/s) | |
|  |  | pre | post | pre | post | pre | post |
| NP | PBS | 65.4±11 | 62±10.0 | 134.1±12 | 113.1±9.1 | 1.2±0.2 | 1.1±0.2 |
|  | 0.25Cabc | 63.1±11 | 53±10.1 | 131.8±11 | 89.6±13.4 | 1.2±0.2 | 1.2±0.2 |
|  | 0.5 Cabc | 63.9±11 | 47.2±9.2 | 130.4±11 | 82.0±11.2 | 1.2±0.2 | 1.2±0.2 |
| iAF | PBS | 40.1±5.1 | 38.1±8.8 | 75.6±6.6 | 74.6±9.4 | 1.2±0.2 | 1.1±0.1 |
|  | 0.25Cabc | 41.0±4.9 | 38.4±9.3 | 74.3±5.8 | 71.2±8.7 | 1.2±0.2 | 1.1±0.2 |
|  | 0.5 Cabc | 40.2±6.3 | 38.6±11 | 76.2±7.1 | 70.1±10.1 | 1.2±0.2 | 1.2±0.2 |
| oAFa | PBS | 25.7±3.3 | 24.7±6.3 | 35.1±5.3 | 34.2±6.5 | 1.2±0.1 | 1.1±0.1 |
|  | 0.25Cabc | 26.1±5.1 | 25.6±7.4 | 36.5±4.8 | 34.5±8.2 | 1.1±0.2 | 1.2±0.2 |
|  | 0.5 Cabc | 25.9±4.1 | 24.8±8.2 | 35.9±3.8 | 33.8±7.6 | 1.1±0.3 | 1.1±0.2 |
| oAFl | PBS | 30.1±4.5 | 28.2±6.2 | 46.8±6.1 | 45.2±6.5 | 1.1±0.3 | 1.1±0.2 |
|  | 0.25Cabc | 28.5±6.1 | 26.3±5.4 | 44.2±5.8 | 44.0±7.2 | 1.1±0.3 | 1.1±0.2 |
|  | 0.5 Cabc | 29.2±5.3 | 27.9±7.4 | 48.6±5.7 | 46.3±5.8 | 1.0±0.2 | 1.1±0.3 |
| oAFp | PBS | 27.1±2.8 | 26.2±6.8 | 41.1±4.7 | 40.6±6.2 | 1.1±0.3 | 1.1±0.2 |
|  | 0.25Cabc | 27.0±3.1 | 25.2±5.3 | 38.2±6.3 | 34.1±5.9 | 1.0±0.2 | 1.1±0.2 |
|  | 0.5 Cabc | 27.3±3.0 | 26.8±8.1 | 39.6±5.4 | 32.5±5.5 | 1.0±0.3 | 1.1±0.2 |

Supplementary table with all pre- and post-experimental MRI values of T2, T1rho, and ADC maps (mean ± SD) per experimental group and disc region. There were no significant differences in values between experimental groups per ROI. Of note: for the statistical analyses in the study the differences (delta values) between paired pre-post measurements per sample within a group were used (and the mean ± standard deviation of these differences) and not overall experimental group means as inter- and intra-individual variation would obscure intervention effects.
